# Utility of recombinant fusion protein ESAT6-CFP10 skin test for differential diagnosis of active tuberculosis: A prospective study

**DOI:** 10.3389/fimmu.2023.1162177

**Published:** 2023-04-25

**Authors:** Yuan Yuan, Lu Xia, Qiaoyu Wu, Xuhui Liu, Shuihua Lu

**Affiliations:** ^1^ Shanghai Public Health Clinical Center, Fudan University, Shanghai, China; ^2^ National Clinical Research Center for Infectious Diseases, The Third People’s Hospital of Shenzhen, The Second Affiliated Hospital of Southern University of Science and Technology, Shenzhen, Guangdong, China; ^3^ Bengbu Medical College, School of Life Science, Bengbu, Anhui, China

**Keywords:** tuberculosis, ESAT6-CFP10 skin test, IGRA, diagnostic accuracy, coherence

## Abstract

**Background:**

The recombinant mycobacterium tuberculosis fusion protein ESAT6-CFP10 skin test (ECST) is a novel test for tuberculosis (TB) infection; however, its accuracy in active tuberculosis (ATB) remains uncertain. This study aimed to evaluate the accuracy of ECST in the differential diagnosis of ATB for an early real-world assessment.

**Methods:**

This prospective cohort study recruited patients suspected of ATB in Shanghai Public Health Clinical Center from January 2021 to November 2021. The diagnostic accuracy of the ECST was evaluated under the gold standard and composite clinical reference standard (CCRS) separately. The sensitivity, specificity, and corresponding confidence interval of ECST results were calculated, and subgroup analyses were conducted.

**Results:**

Diagnostic accuracy was analyzed using data from 357 patients. Based on the gold standard, the sensitivity and specificity of the ECST for patients were 72.69% (95%CI 66.8%-78.5%) and 46.15% (95%CI 37.5%-54.8%), respectively. Based on the CCRS, the sensitivity and specificity of the ECST for patients were 71.52% (95%CI 66.4%-76.6%) and 65.45% (95%CI 52.5%-78.4%), respectively. The consistency between the ECST and the interferon-γ release (IGRA) test is moderate (Kappa = 0.47).

**Conclusion:**

The ECST is a suboptimum tool for the differential diagnosis of active tuberculosis. Its performance is similar to IGRA, an adjunctive diagnostic test for diagnosing active tuberculosis.

**Clinical trial registration:**

http://www.chictr.org.cn, identifier ChiCTR2000036369.

## 1 Introduction

In 2021, ~10.6 million people worldwide were newly infected with tuberculosis (TB), whereas the number of newly diagnosed TB was only 6.4 million (1). To achieve the World Health Organization’s goal of ending TB by 2035, it is essential to screen and diagnose TB. Until now, the detection methods for diagnosing TB are limited. Tuberculin skin test (TST) and interferon-γrelease (IGRA) test are the main methods for screening TB; TST has poor specificity and IGRA is expensive and requires specialized laboratory conditions. Therefore, a new diagnostic method with high specificity and low cost is urgently needed.

Recombinant *Mycobacterium tuberculosis* fusion protein ESAT6-CFP10 (EC) skin test was made from the recombinant *Mycobacterium tuberculosis* fusion protein obtained by *Escherichiacoli* after fermentation, isolation, and purification (2, 3). Compared with the IGRA test, the ESAT6-CFP10 skin test (ECST) is performed by intradermal injection of recombinant ESAT6-CFP10 antigen. The EC test solves the false-positive problem after BCG vaccination and combined the advantages of the TST and the high specificity of IGRA detection. At present, EC (trade name: YiKa) has obtained the national first-class new drug and completed clinical trials on April 23, 2021.

The sensitivity, specificity, and safety of diagnostic reagents are concerns in clinical diagnosis. The sensitivity, specificity, and safety of ECST were evaluated by phase I, II, and III clinical trials before marketing. However, phase III clinical trial participants were strictly screened and did not include those with underlying diseases and comorbidities. Owing to the lack of ECST results in TB patients with comorbidities, this study aimed to evaluate the accuracy and safety of the ECST in tertiary hospitals to assess its value in the diagnosis of ATB and provide data support for the subsequent large-scale post marketing re-evaluation.

## 2 Methods

### 2.1 Study design and participants

A prospective cohort study was conducted in Shanghai Public Health Clinical Center from January 2021 to November 2021. All participants suspected pulmonary TB (PTB) were consecutively recruited from inpatient services. Including routine laboratory examinations, each participant would receive IGRA (T-SPOT.TB. Oxford, UK.) and ECST. The results were compared by statistical analysis. The primary outcome of this study was a comparison of the diagnostic accuracy of ECST and T-SPOT.TB assays for active TB. The secondary outcomes included the consistency between the two assays, the diagnostic yields in different subgroups, and the safety of ECST.

### 2.2 Study procedure

**Figure 1:**
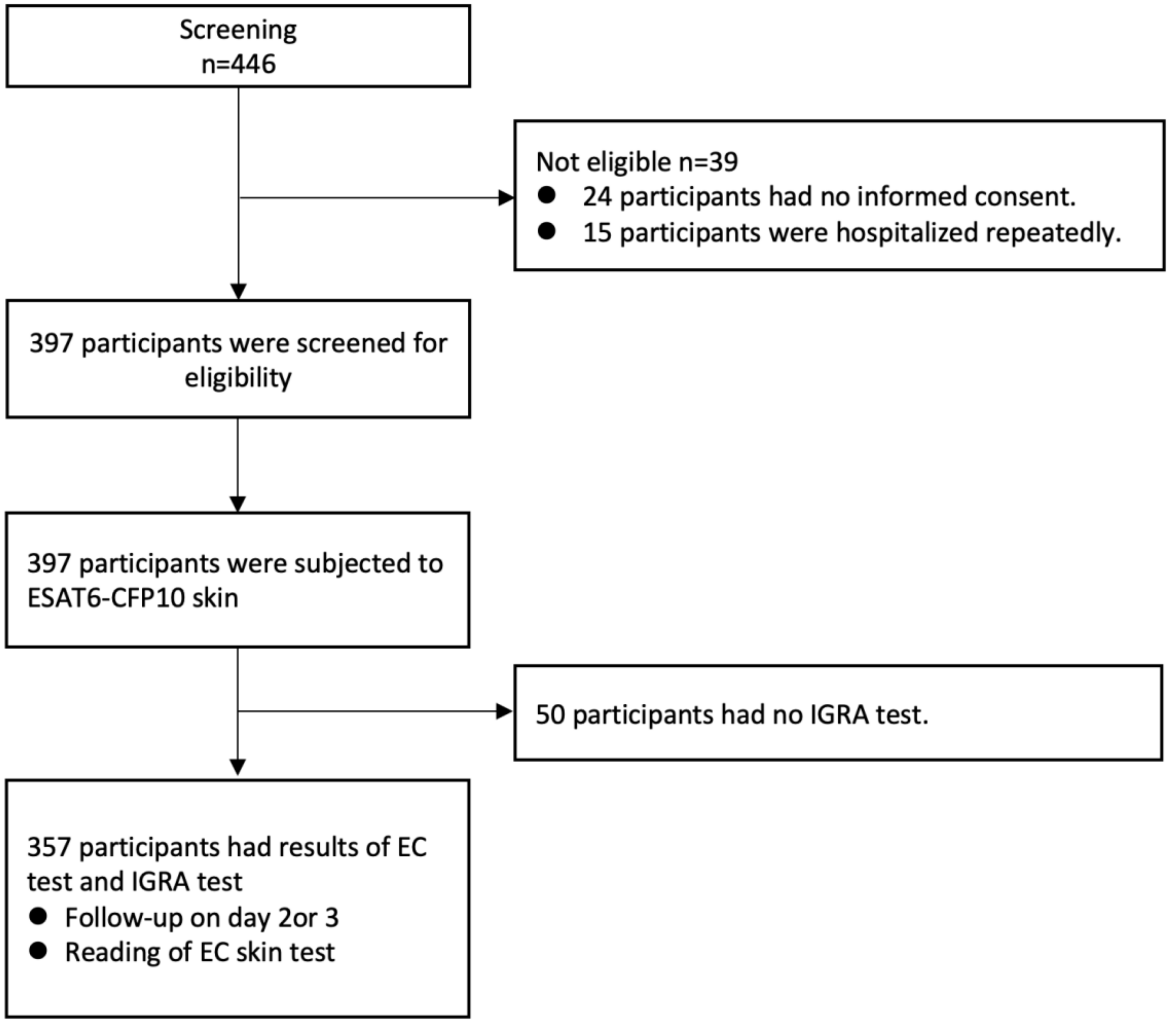
Study flow diagram.

From January to November 2021, the accuracy of the ECST was evaluated in the Tuberculosis Department of Shanghai Public Health Clinical Center. All participants underwent an IGRA test and then an ECST (**Figure 1**). The transverse and longitudinal diameters of skin erythema and/or induration at the injection site were measured in millimeters at different time points. Skin erythema was defined as visible red discoloration of the skin at the injection site, and induration was measured by palpation of the forearm. Systemic and local adverse events were recorded within 72 h of injection. Systemic and local adverse events, such as rash, pain, and itching, and adverse reactions such as anaphylactic shock, local tissue ulcer, local necrosis and liquefaction, systemic allergic rash, systemic urticaria, and allergic purpura, were observed and recorded. An adverse event was defined as any adverse event in a patient who underwent the ECST.

### 2.3 Statistical analysis

ECST results were expressed as the number of millimeters of the transverse and longitudinal diameters of erythema or induration at the forearm injection site at 48–72 h after the ECST injection. The results were based on the redness or induration, and the average diameter of the reaction (sum of the horizontal and vertical diameters divided by 2) was not less than 5 mm. Those with blisters, necrosis, and lymphangitis were strongly positive.

IBM SPSS Statistics version 26.0 (IBM Corp., Armonk, NY, USA) was used as a statistical tool. The clinical diagnosis results and bacteriological results were taken as reference standards to calculate the sensitivity and specificity of the EC reagent and IGRA detection, draw the ROC curve, and assess the consistency between the ECST and the IGRA test. The chi-square test was used to compare categorical variables, and the t-test was used to analyze continuous variables. 95% Confidence intervals were calculated based on bilateral distribution. All statistical tests were two-tailed, with P < 0.05 as the significant difference.

### 2.4 Case definitions and inclusion/exclusion criteria

Active PTB patients were diagnosed in concordance with the diagnostic standard by the National Health Commission of the People’s Republic of China (4), The following criteria will increase the possibility for making a PTB diagnosis: (1) house-hold tuberculosis contact in the prior 3 months, (2) fever or cough for more than two weeks, weight loss or failure to gain weight in the previous 3 months, (3) a positive tuberculin skin test or interferon-γ release assay result, (4) a chest radiograph suggestive of TB. (5) effective anti-tuberculosis treatment, (6) smear positive on respiratory tract specimen with/without positive culture or xpert assay. Patients who met the above criteria were bacteriologically confirmed TB, patients who met 1-5 criteria except the sixth criterion were clinically diagnosed TB. Microbiological reference standard of PTB was defined as the gold standard.

Suspected pulmonary TB cases were defined as those who aroused a suspicion of pulmonary TB but for whom a clinical diagnosis and decisions were not made.

Non-TB is a patient who does not meet the criteria for the clinically diagnosed tuberculosis.

The inclusion criteria were as follows: (1) age > 6 months, (2) suspected tuberculosis, and (3) willing to provide written informed consent.

The exclusion criteria were as follows: (1) pregnancy or lactation, (2) children with congenital immunodeficiency, (3) HIV infection, (4) live vaccination or biological agent within 4 weeks, (5) previous mental illness or cognitive dysfunction, and (6) other circumstances that the researcher considered unsuitable for the experiment.

## 3 Results

### 3.1 Participant characteristics

A total of 446 patients were screened for this study, excluding 24 who were unwilling to sign the informed consent form, 15 with repeated admissions, and 50 without IGRA test results. All the enrolled patients were suspected TB, and 50 of them did not have IGRA test results. These 50 patients were TST-positive and did not want to be tested for IGRA. A total of 357 patients underwent the EC skin and IGRA tests, of which 302 were clinically diagnosed with TB and 55 were not diagnosed with TB. There were 247 male and 110 female patients. The youngest and older patients were 2 and 94 years old, respectively. The mean age was 51.50 and 54.00 years old in the TB and non-TB groups, respectively, and no significant difference was noted between the two groups (P = 0.96). The mean body mass index values of the two groups were 20.87 and 20.78 kg/m^2^, respectively, and no significant difference was found between the two groups (P = 0.13) (**Table 1**).

**Table 1:** Clinical characteristics of the participants in the tuberculosis and non-tuberculosis groups.

	TB	Non-TB	P value
	(N = 302)	(N = 55)	
Age, years (IQR)	51.50 (31.0–65.0)	54.00(27.00-68.00)	0.96
Female, sex, n. (%)	96(31.79)	14(25.5)	0.35
BMI, mean (kg/m^2^)	20.87 ± 3.52	20.78 ± 4.19	0.13
Cancer, n. (%)	8 (2.64)	3 (5.45)	0.49
Hypertension, n. (%)	62 (20.53)	9 (16.36)	0.48
Diabetes, n (%)	57 (18.87)	9(16.40)	0.66
Hyperlipemia, n (%)	6 (1.98)	2 (3.64)	0.79
Liver disease, n (%)	46 (15.23)	11 (20.00)	0.38
Nephropathy, n (%)	22 (7.28)	6 (10.91)	0.52
Anemia, n (%)	25 (8.28)	4 (7.27)	1.00
Connective tissue disease, n. (%)	6 (1.98)	1 (1.82)	1.00
Bacteriological diagnosed tuberculosis, n(%)	161	5	0.00
Extrapulmonary tuberculosis	69	0(0.00)	0.00
Pass infected tuberculosis n. (%)	4 (0.01)	14(25.5)	0.00

### 3.2 Sensitivity and specificity of the ECST

Based on the gold standard, 227 patients comprised the TB group and 130 made up the non-TB group. The sensitivity of the ECST was 72.69% (66.8%-78.5%), the specificity was 46.15% (37.5%-54.8%), and the AUC_EC_ was 0.59. The sensitivity and specificity of IGRA were 86.34% (81.8%-90.8%) and 36.15% (27.8%-44.5%), respectively, and the AUC_IGRA_ was 0.61 (**Table 2**).

**Table 2:** Comparison of the accuracy of the ECST and IGRA tests in patients with bacteriological diagnosed tuberculosis.

	Result	TB (n)	Not TB (n)	Sensitivity (%)	95%CI	Specificity (%)	95%CI	AUC
ECST	+	165	70	72.69	66.8-78.5	46.15	37.5-54.8	0.59
	–	62	60					
IGRA	+	196	83	86.34	81.8-90.8	36.15	27.8-44.5	0.61
	–	31	47					

**Figure 2:**
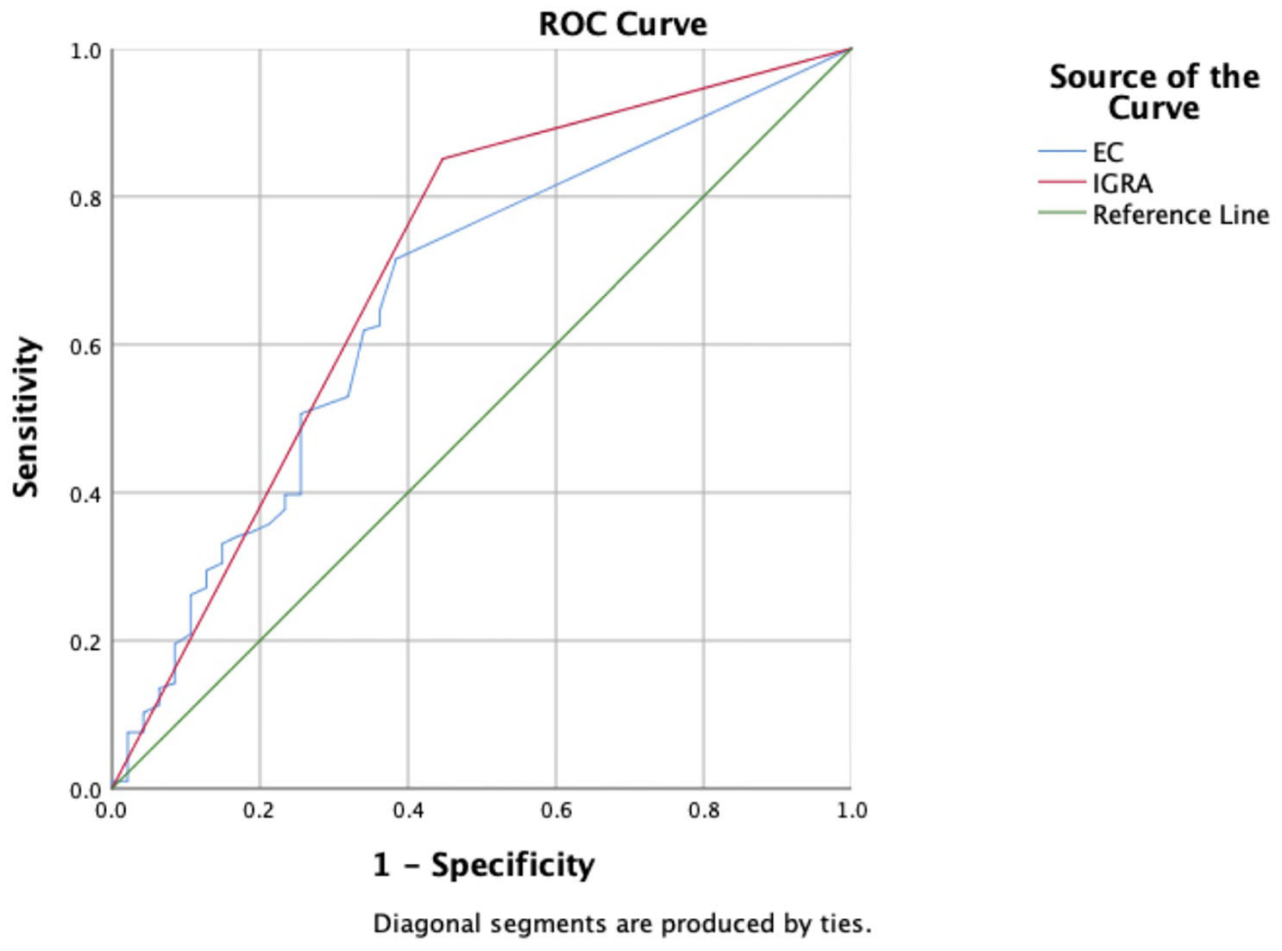
Receiver operating characteristic curves of the ECST and IGRA tests in patients with clinically diagnosed tuberculosis.

Based on composite clinical reference standard (CCRS), 302 patients had TB and 55 patients do not have TB. The sensitivity of the ECST was 71.52% (66.4%–76.6%), the specificity was 65.45% (52.5%-78.4%), and the AUC_EC_ was 0.69. The sensitivity and specificity of the IGRA test were 85.10% (81.1%–89.1%) and 60.00% (46.6%-73.4%), respectively, and the AUC_IGRA_ was 0.73 (**Table 3**, **Figure 2**).

**Table 3:** Comparison of the accuracy of the ECST and IGRA tests in patients with clinically diagnosed tuberculosis.

	Result	TB (n)	Not TB (n)	Sensitivity (%)	95% CI	Specificity (%)	95%CI	AUC
ECST	+	216	19	71.52	66.4–76.6	65.45	52.5-78.4	0.69
	–	86	36					
IGRA	+	257	22	85.10	81.1–89.1	60.00	46.6-73.4	0.73
	–	45	33					

### 3.3 Subgroup analysis

In the baseline analysis, a history of TB was statistically different between the TB group and the non-TB group (**Table 1**). After the subgroup analysis, the sensitivity of the ECST was 72.73% (66.7%–77.7%), the specificity was 73.17% (59.0%-87.3%), and the AUC_EC_ was 0.73. The sensitivity and specificity of the IGRA test were 85.57% (81.6%–89.6%) and 68.29% (53.4%-83.2%), respectively, and the AUC_IGRA_ was 0.77 (**Table 4**).

**Table 4:** Comparison of the accuracy of ECST and IGRA tests in newly diagnosed TB.

	Result	TB(n)	Not TB(n)	Sensitivity (%)	95%CI	Specificity (%)	95%CI	AUC
ECST	+	224	11	72.73	67.7–77.7	73.17	59.0-87.3	0.73
	−	84	30					
IGRA	+	255	13	85.57	81.6–89.6	68.29	53.4-83.2	0.77
	−	43	28					

**Figure 3:**
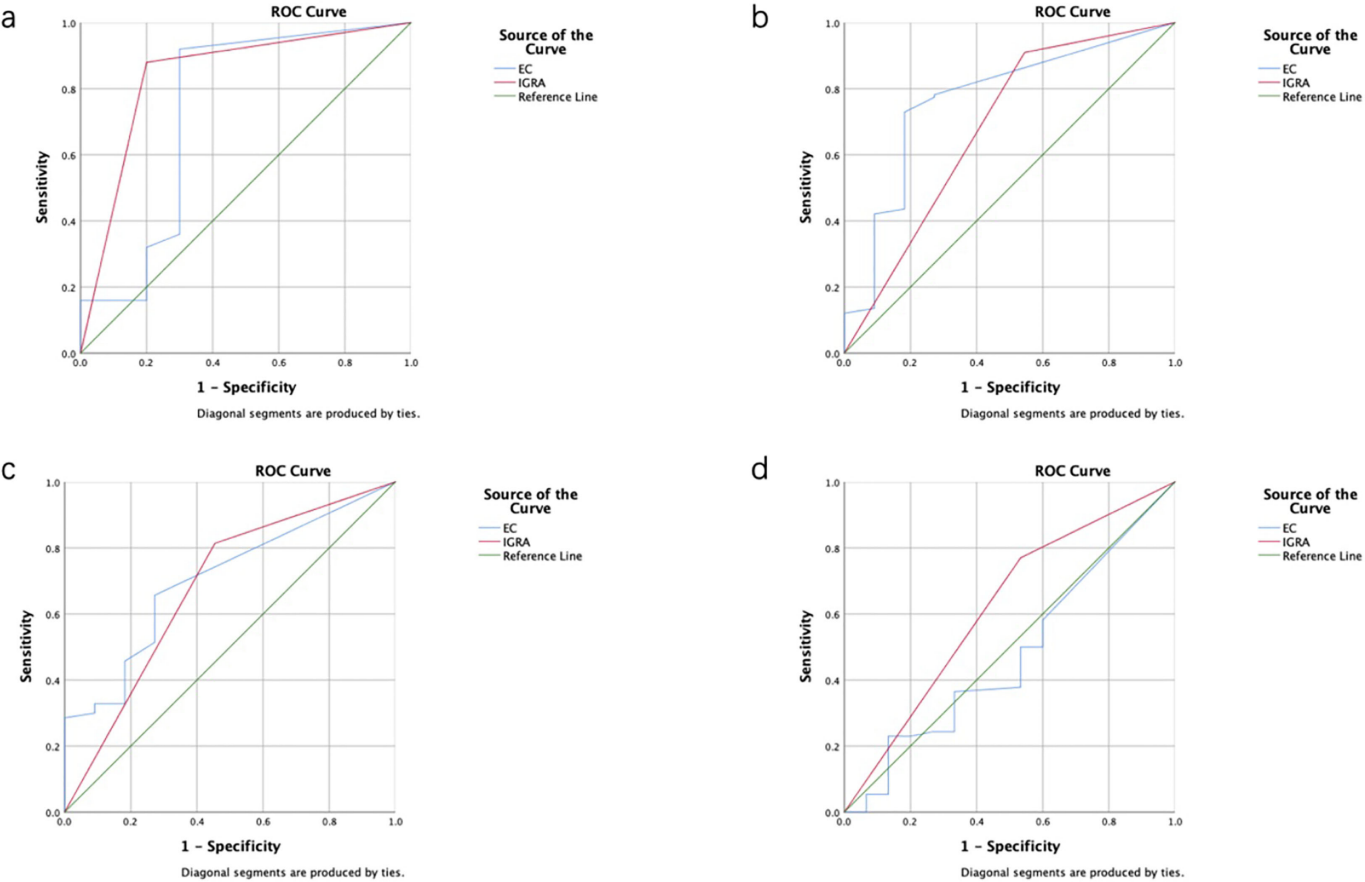
Receiver operating characteristic (ROC) curves of the ECST and IGRA tests in patients of different ages. **(A)** ROC curves for EC skin and IGRA tests in patients aged 0–18 years. **(B)** ROC curves for EC skin and IGRA tests in patients aged 19–52 years. **(C)** ROC curves for EC skin and IGRA tests in patients aged 52–65 years. **(D)** ROC curves for EC skin and IGRA tests in patients aged 65–94 years.

Considering that patient age may affect the sensitivity and specificity of the EC skin and IGRA tests, analyses of different age groups were performed. The sensitivity and specificity of the ECST were 88.00% and 72.73%, in patients aged 0–18 years, 77.44% and 73.33% in patients aged 19–52 years, 68.57% and 72.73% in patients aged 52–65 years, and 58.11% and 50.00% in patients aged 65–94 years, respectively (**Table 5**). The sensitivity and specificity of the IGRA test were 88.00% and 81.82% for patients aged 0–18 years, 90.98% and 53.33% for patients aged 19–52 years, 81.43% and 54.55% for those aged 52–65 years, and 77.03% and 55.56% for those aged 65–94 years, respectively (**Table 6**). The ROC curves were drawn for participants of different ages (**Figure 3**), 36 patients were between 0 and 18 years old, and the AUC_EC_ and AUC_IGRA_ were 0.80 and 0.85, respectively. Moreover, 148 patients were 19–52 years old, and the AUC_EC_ and AUC_IGRA_ were 0.75 and 0.72, respectively. In addition, 81 patients were 52–65 years old, and the AUC_EC_ and AUC_IGRA_ were 0.71 and 0.68, respectively. There were 92 patients aged 65–94, and the AUC_EC_ and AUC_IGRA_ were 0.54 and 0.66, respectively (**Tables 5**, **6**).

**Table 5:** Comparison of the accuracy of the ECST in patients of different ages with clinically diagnosed tuberculosis.

ECST	Result	TB (n)	Not TB (n)	Sensitivity (%)	95% CI	Specificity (%)	95%CI	AUC
0–18	+	22	3	88.00	74.3–101.7	72.73	41.3-104.1	0.80
	−	3	8					
19–52	+	103	4	77.44	70.2–84.6	73.33	48.0-98.7	0.75
	−	30	11					
52–65	+	48	3	68.57	57.4–79.7	72.73	41.3–104.1	0.71
	−	22	8					
65–94	+	43	9	58.11	46.6–69.6	50.00	24.4-75.6	0.54
	−	31	9					

**Table 6:** Comparison of the accuracy of IGRA test in patients of different ages with clinically diagnosed tuberculosis.

IGRA	Result	TB (n)	Not TB (n)	Sensitivity (%)	95% CI	Specificity (%)	95% CI	AUC
0–18	+	22	2	88.00	74.3–101.7	81.82	54.6-109.0	0.85
	−	3	9					
19–52	+	121	7	90.98	86.0–95.9	53.33	24.7-81.9	0.72
	−	12	8					
52–65	+	57	5	81.43	72.1–90.8	54.55	19.5–89.6	0.68
	−	13	6					
65–94	+	57	8	77.03	67.2–86.8	55.56	30.1-81.0	0.66
	−	17	10					

### 3.4 Consistency of EC skin and IGRA tests

In all patients, the kappa value of the ECST and the IGRA test was 0.47. Consistency analysis was conducted for patients with TB at different sites, and the kappa values were 0.29 and0.54 in patients with TB and simple extrapulmonary TB, respectively. The kappa values of different age groups were 0.66, 0.46, 0.40 and 0.35.

### 3.5 Safety evaluation of the ECST

Grade 3 and 4 adverse reactions were not observed. A total of 33 (9.24%) patients had grade 1–2 adverse reactions, and the most common complaints were pain and pruritus at the injection site. Among them, 18 were men and 15 were women. Local adverse reactions occurred in two children, 14 patients aged 19–44 years, 16 patients aged 45–74 years, and 1 patient aged >74 years.

## 4 Discussion

In China, suspected pulmonary TB patients with complex conditions, comorbidities, and uncertain diagnoses often seek treatment in tertiary hospitals. Since China is an area with high prevalence of *Mycobacterium tuberculosis*, the proportion of people with *Mycobacterium tuberculosis* infection among non-TB patients may be as high as 20%. If the patient is combined with immunocompromised status or other special diseases, such as silicosis, end-stage renal disease, HIV infection, etc., the risk of tuberculosis infection will be even higher. These patients may yield positive ECST results despite no active TB, theoretically. Therefore, we tested the differential diagnostic value of ECST in an environment with many interfering factors (a real-world setting), and this can be viewed as a stress testing of ECST. In this study, all patients underwent ECST. In a previous phase III clinical trial, we demonstrated the consistency and safety of ECST in healthy individuals and patients with TB. In the present clinical trial, we focused on evaluating the accuracy of ECST in the differential diagnosis of active TB in tertiary specialized hospitals.

The study showed no significant difference in the sensitivity of ECST among patients with bacteriological diagnosed TB (72.69%), clinically diagnosed TB (71.52%), and newly diagnosed TB (72.73%). Meanwhile, the specificity of ECST in clinical diagnosis was 65.45%; however, in the subgroup, the specificity of the ECST in newly diagnosed TB was 73.17%, which indicated that the diagnostic efficacy was higher after excluding TB history. The specificity of the IGRA test in this trial was comparable, i.e., 60.00% vs. 68.29%, which is similar to the result of a previous study in which the specificity of the IGRA test was lower in patients with a history of TB than in patients without a history of it (21% [9%–39%] vs. 63% [55%–71%], P = 0.001) (5).

The sensitivity and specificity of Diaskintest were 78.1%–88.9% and 92.1%–96.4% (6, 7), and that of C-Tb were 73.9% (95% CI 67.8–79.3) and 99.3%, respectively (8, 9). The ECST produced by Anhui Zhifeilong Koma Biological Co., Ltd., showed a sensitivity of 87.5% (77.8%–97.2%) and specificity of 98.4% (95.4%–99.7%) in a phase II b clinical trial (10). In a phase III clinical trial, the 48-h sensitivity and specificity of ECST were 90.85% and 89.83%, respectively (11). The sensitivity and specificity of this test were 71.52% and 65.45%, respectively. The factors that affect the results of skin tests include product type, quality, and dose. Infection was related to the immune status, age, and physical strength of the vaccinated participants. Moreover, it is related to the inoculation technique by the medical staff and the evaluation technique of the results. The positive rate of ECST in this study was low because both stage II and stage III were RCTS, and highly homogeneous participants were selected through a series of nanoscale criteria. Patients who had a challenging diagnosis and had more underlying diseases were found in tertiary specialized hospitals. In this study, most of the hospitalized patients were not first-time patients, and most of them have atypical TB, or multidrug-resistant TB, which is difficult to diagnose. Even though all patients had TB, large individual differences exist and many had complicated diseases. In this study, the patients mainly had 3–5 diseases, 235 (65.8%) were >45 years old, 71 (19.9%) had hypertension, 66 (18.5%) had diabetes, and 28 (7.84%) had kidney disease among common diseases.

In the present study, the AUC value of the ECST was close to that of the IGRA test (0.59 vs. 0.61), indicating little difference in accuracy. However, the kappa value was only 0.47, indicating that the critical value of the ECST set at 5 mm in tertiary specialized hospitals may not be appropriate and should be further evaluated. In a phase IIb trial, the consistency of EC skin and IGRA tests was good, possibly because the left forearm of IIb received 0.1 mL of TB-PPD, and the right forearm received 0.5 µg/0.1 mL or 1.0 µg/0.1mL in the ECST; however, only 0.1 mL was injected in this trial. The low AUC and kappa values may be attributed to the different doses of the antigen injected.

In this study, 61 (15.7%) patients had EC-negative and IGRA-positive findings, including 45/61 (73.8%) patients aged >45 years, possibly due to aging or immunosuppression, which is consistent with the conclusion that accuracy decreases with age in the subpopulation. The IGRA detection in the present study also showed a similar trend, consistent with the conclusion in previous studies that the sensitivity of IGRA detection in patients with immunocompromised status was lower than that in patients with normal immune function (53% [29%–76%] vs. 83% [67%–92%], P = 0.045). Therefore, for patients of different ages and different underlying diseases, especially those with low immunity, 5 mm as the threshold value may be too general. We also found elder age had obviously impact on ECST rather than IGRA. By comparing the results at all ages, the sensitivity of ECST decreased from 88% to 58.11%, while the sensitivity of IGRA detection decreased by only 11%. Age may be an influencing factor. This result may also be due to the small sample size in the sub group analysis. what’s more, the specificity of IGRA detection was only 55.56%, which may also be due to the sample size. Clinical trials with larger sample sizes will be needed to verify this conclusion.

This study has some limitations. First, immunological test results may be affected before and after treatment. In real-world tests, auxiliary diagnosis is usually performed before treatment or within 2 weeks after treatment. TB screening is recommended at the first test or 3 months after the first test. Second, the patients in this study lacked a TB treatment history for evaluation. Third, as patients aged <65 years were included in phases II and III, patients aged >65 years in the present study were not well compared with previous studies. Fourth, this study did not record in detail whether the patient had received TST at the first hospital and whether there was an enhancement effect after receiving the ECST again. Fifth, the experiment was conducted in the TB department, where most patients had TB; thus, the proportions of the TB and non-TB groups were unreasonable. The diagnostic accuracy and safety of ECST in tertiary specialized hospitals are average; thus, follow-up studies with larger populations are needed.

## 5 Conclusion

The ECST is a suboptimum tool for the differential diagnosis of active tuberculosis. Its performance is similar to IGRA, an adjunctive diagnostic test for diagnosing active tuberculosis.

## Data availability statement

The original contributions presented in the study are included in the article/supplementary material. Further inquiries can be directed to the corresponding authors.

## Ethics statement

This trial was conducted in compliance with the Declaration of Helsinki and principles of Good Clinical Practice. The studies involving human participants were reviewed and approved by the ethics committee of Shanghai Public Health Clinical Center (No.2020-S121-01). Written informed consent to participate in this study was provided by the participants' legal guardian/next of kin.

## Author contributions

SL, XL, and LX conceived and designed the study. YY and QW were involved in data analysis and collection. YY drafted and wrote the article and all authors provided critical revisions. All authors contributed to the article and approved the submitted version.
